# Antibacterial activity of honeys and potential synergism of honeys with antibiotics and alkaloid extract of *Sophora alopecuroides *plant against antibiotic-resistant *Escherichia coli *mutant

**DOI:** 10.22038/ijbms.2021.54224.12179

**Published:** 2021-05

**Authors:** Razieh Pourahmad Jaktaji, Farideh Ghalamfarsa

**Affiliations:** 1 Department of Genetics, Faculty of Science, Shahrekord University, Shahrekord, Iran

**Keywords:** AcrAB-TolC, Alkaloid extract, Escherichia coli, Honey, Sophora alopecuroides, SoxS

## Abstract

**Objective(s):**

The increase in multidrug-resistant *Escherichia coli *strains with an overactivated AcrAB-TolC efflux pump has reduced the effectiveness of synthetic antibiotics, such as ciprofloxacin. The activity of this efflux pump can be reduced by using natural products. This study aimed to use a combination of ciprofloxacin, honey, and alkaloid extract of *Sophora alopecuroides* against an *E. coli* mutant with an overactivated AcrAB-TolC pump.

**Materials and Methods:**

First the physicochemical properties, total alkaloid content, antioxidant activity, and the minimum inhibitory concentration (MIC) of three local honey samples: Konar (lotus), Avishan (Thyme), and Gavan (Astragalus) were evaluated. Then, the MIC of different combinations of honey, ciprofloxacin, and plant alkaloid extract and expression of *acrA* and *soxS* genes were carried out using the agar dilution method and quantitative RT- PCR methods.

**Results:**

The net absorbance, total alkaloid content, and DPPH radical scavenging activity of Konar honey were significantly higher than those of Avishan and Gavan honeys (*P*<0.05). However, the MIC of lotus honey was nearly similar to other honey types, and all honey (30% w/v)-ciprofloxacin combinations decreased the viability of mutant more than ciprofloxacin alone. A synergistic interaction (FICI =0.48) was observed in triplex complex of ciprofloxacin (10 µg/ml), honey (20% w/v), and plant extract (1 mg/ml). A significant decrease (*P*<0.05) in the expression level of genes was seen in the presence of the triplex complex.

**Conclusion:**

It is concluded that the interaction between honey and plant alkaloid extract enhanced the anti-pump activity and reduced the oxidative stress response of the *E. coli *mutant.

## Introduction

Honey is a viscous liquid made by honeybees from the nectar of flowers (nectar honeys) or exudates of trees and plants (honeydew honeys). Honey is also classified by its floral source into monofloral and polyfloral honey. Monofloral honey is produced from the nectar of various plants, while the nectar of one single species is predominant (1, 2). Avishan, Gavan, and Konar honeys are typical examples of monofloral honey produced in Iran. 

Honey has nutritional values and numerous health benefits, such as healing wounds. The nutritional value and health benefits of honey are related to its antibacterial activity (3). On the other hand, the antibacterial activity of honey is dependent on many factors, including acidity (low pH of honey), osmolarity (high sugar content of honey), and presence of hydrogen peroxide (generated by glucose oxidase), anti-oxidants (phenolic acids and flavonoids), defensin-1, and methylglyoxal (MGO) (3). 

Manuka honey is well-characterized honey, derived from the manuka tree (from the genus *Leptospermum*), native to New Zealand and Australia. This type of honey has been registered as a wound care product. The amount of MGO differs in various *Leptospermum* species from about 100 ppm to more than 1200 ppm. MGO can react non-specifically with macromolecules, including DNA, RNA, and proteins (4, 5). 

Additionally, the amount of hydrogen peroxide varies in different types of honey, depending on the floral source and environmental factors. The inhibitory concentration of hydrogen peroxide against bacteria may be higher than its concentration found in honey. However, honey without this constituent still shows antibacterial activity, which is known as non-peroxide activity (NPA). NPA is mostly related to MGO and various phenolic compounds, which act as anti-oxidants in manuka honey. Although the levels of these compounds are too low to be inhibitory alone, it was found that these compounds may synergize with one another or other constituents of honey to produce or alter the activity (3, 4). 

With the introduction of a variety of antibiotics in the market, the clinical use of honey was abandoned. However, with the emergence of resistance to antibiotics worldwide, the potent activity of honey (manuka and local honeys) against a broad range of antibiotic-resistant bacteria was investigated (5-7). The growth-inhibitory effect of honey was demonstrated on different Gram-positive (*Staphylococcus aureus* and *Streptococcus pyogenes*) and Gram-negative (*Escherichia coli*) bacteria (5, 8). Nevertheless, there was no reduction in the sensitivity of clinical isolates with multiple drug-resistant (MDR) phenotypes to honey (5-8). 

Honey has been used along with different antibiotics to augment their potency against pathogenic bacteria. The synergistic effect of honey with oxacillin, tetracycline, imipenem, and mupirocin on methicillin-resistant *S. aureus* strains (MRSAs) has been reported in the literature (7, 8). 

Ciprofloxacin (fluoroquinolone antibiotic) stimulates the production of reactive oxygen species (ROS), including superoxide and hydroxyl radicals in bacterial cells (9). Bacteria contain defense systems against DNA damage caused by ROS, including SOS and SoxRS regulons. Activation of these regulons confers resistance to multiple drugs through overexpression of the AcrAB-TolC efflux pump. 

Honey may show synergistic effects with ciprofloxacin (CIP), against MDR strains of *E. coli*. The synergistic interaction between CIP and total alkaloid extract of local *Sophora alopecuroides* (TASA) has been reported against the MDR strain of *E. coli* with the overexpression of AcrAB-TolC efflux pump (10). It was found that TASA could not completely suppress the pump activity. This study aimed to examine the interaction of three monofloral types of honey with CIP and TASA against an MDR strain of *E. coli*. 

## Materials and Methods

***Chemicals, bacterial strains, and honey samples***

Antimicrobial compounds (ciprofloxacin and tetracycline), DPPH (2.2-diphenyl-picrylhydrazyl hydrates radical), ascorbic acid, and atropine were purchased from Sigma (St Louis, MO, USA). Luria–Bertani (LB) broth, agar, Mueller–Hinton agar, methanol, chloroform, and bromocresol green (BCG) were from Merck (Darmstadt, Hesse, Germany). Strains used in this study (MG1655 and M1) were described previously (10). Briefly, MG1655 is a wild-type strain and M1 is a* gyrA marR* double mutant with high resistance to ciprofloxacin. Three samples of monofloral honeys (Avishan, Gavan, and Konar) were bought in the year 2016 from a local shop and stored at room temperature in dark until the time of analysis. Avishan honey (Thyme) was produced in the Kangavar region in Kermanshah Province from mostly Thymus kotschyanus Boiss L. This is a small shrub with sharp leaves and white flowers that belongs to the family *Lamiaceae *(11). Konar honey (lotus) was made in Haj Abad area in Bushehr Province from predominantly Ziziphus spina-christi L. This is an evergreen tree with dense prickly branches, bright oval leaves, small white star flowers, and small circular fruit, which belongs to the family *Rhamnaceae * (12). Gavan honey (Astragalus) was produced in the Kohrang region in Chahar Mahal va Bakhtiari Province from mostly *Astragalus verus *Olivier. This is a small vascular thorny shrub with yellow flowers that belongs to the family *Fabaceae* (13). 

***Determination of pH and acidity***

10 g of honey sample was dissolved in 75 ml of carbon dioxide-free water and the pH was measured using a pH meter 3310 (Jenway, UK). Then the honey sample was titrated with 0.1 M NaOH to pH 8.3. Free acidity, was expressed as milliequivalents acid (meq)/kg honey= ml 0.1 M NaOH˟10 [1]. The assay was conducted three times on honey samples. 

***Colorimetric test for flavonoids and Net absorbance (color intensity)***

To determine the presence of flavonoids in honey samples, ammonia solution (1N) was added to a portion of honey samples followed by addition of concentrated H_2_SO_4_. A yellow coloration indicates the presence of flavonoids (14). Since three samples of honey showed the presence of flavonoids, the net absorbance of honey samples was measured three times by UV-visible spectrophotometry (Ultrospec 1100, Amersham Pharmacia Biotech) as explained before (1,15). Briefly, honey samples were diluted to 50% (w/v) with warm (45–50 °C) sterile double distilled water. The absorbance was measured at 450 and 720 nm and the difference between absorbance measurements at 450 and 720 nm was expressed as AU (absorbance unit). 

***Total alkaloid content assay***

TASA (total alkaloid extract of *S. alopecuroides*) was prepared as described previously (10). Powdered seeds were macerated in ethanol and then filtered. After evaporation, the concentrate was treated with hydrochloric acid and ammonia and dissolved in chloroform. After evaporation, the dried extract was collected, weighed, and dissolved in distilled water. The presence of alkaloids was verified by Bromocresol Green Solution (BCG) indicator (1˟10^-4^ mol/L), phosphate buffer (pH 4.7), and Atropine standard solution (0.1 mg/ml) as described previously (16). Briefly, the extract was dissolved in 2N HCL and filtered. One ml of this solution was washed with 10 ml chloroform. The pH of this solution was adjusted to neutral with 0.1 N NaOH. Then BCG and phosphate buffer were added and the complex was extracted with different volumes of chloroform in separate flasks. The absorbance of solutions was measured at 417 nm. This assay was performed three times on each sample of honey. 

***DPPH free radical-scavenging activity***

The anti-oxidant property of each honey sample and TASA were studied by evaluating the free radical-scavenging activity of the DPPH radical, which was based on the method explained by Vela *et al. *(1) with minor modification. Briefly, 1.25 ml of honey or TASA solution in sterile double distilled water (ranging from 0.01 to 0. 2 g/ml) was mixed with 1.5 ml of a 0.009% solution of DPPH in methanol. After a 5 min incubation period at dark conditions, the absorbance was read at 517 nm against a water/methanol (1:1 v/v) blank. All determinations were performed in triplicate. 

***Minimum inhibitory concentration (MIC) of materials***

Honey MICs were determined by the agar dilution method as guided by the Clinical and Laboratory Standards Institute (17). Overnight *E. coli* cultures were diluted (1:100) in LB and grown till the exponential phase. These fresh cultures were used to prepare an inoculum of approximately 10^4^ to 10^5^ CFU per spot (10 µl) on Mueller–Hinton agar. Honey concentrations were from 1 to 80% and mixed with the warmed medium before pouring in the plate. Plates were incubated at 37 °C for 20 hr. The number of colonies formed per spot was counted. The MIC of CIP–honey and CIP–honey-TASA combinations was also determined by the agar dilution method. Subinhibitory concentrations of plant extract (1–6 mg/ml), CIP (10 to 60 µg/ml) for resistant clone, and 8–16 ng/ml for the wild-type strain honeys (30–50%) were used for the assay. The experiments were conducted three times. 

***Fractional inhibitory concentration index (FICI)***

FICI was used to show the type of interaction between substances in combination. FICIs for CIP–honey, tetracycline-honey, CIP–honey-TASA, and honey-TASA combinations were calculated according to the formula described previously (18). 

***Bactericidal activity of honey and CIP-honey combination***

Fresh precultured bacteria were diluted (1:100) in Mueller–Hinton broth and grown at 37 °C to OD 0.6. Then cultures were serially diluted and plated on Mueller–Hinton agar containing various concentrations of CIP with or without 30% honey and incubated at 37 °C. The number of colonies formed was counted and expressed as log CFU/ml (19). 

***Total bacterial RNA extraction***

 LB cultures at an A_600_ of 0.6 from the untreated wild-type strain and high resistant clone with three different treatments: single Cip, duplex combination (Cip-honey), and triplex combination (Cip-honey-TASA) were harvested for RNA extraction using an RNeasy Mini Kit (QIAGEN, Hilden, Germany). DNA contamination was removed using RNase-free DNase I (Fermentas, Waltham, MA). cDNA synthesis was performed using a RevertAid Reverse Transcription Kit (Fermentas) with 500 ng of RNA and random hexamer (10). 

***Gene expression analysis by real-time PCR***

 cDNA was used for gene expression by real-time PCR using an SYBR Green Kit (TaKaRa, Otsu, Japan) and a Rotor-Gene 6000 Thermocycler (Corbet Research, Sydney, Australia). Specific primers for *acrA* were explained in a previous study [10] and for *soxS* were: forward primer, 5́ - CCAGGTCCATTGCGATATCA -3′and reverse primer, 5 – CGCATGGATTGACGAGCATA– 3′(product size 201 base pair). Thermal conditions were as described previously (10). 

***Statistical analysis***

Paired Student’s t-test was used for comparison of relative gene expression and other data using SPSS v.16 software (SPSS Inc., Chicago, IL, USA). A significance level of *P*<0.05 was chosen in the statistical analyses. Gene expression equal to 2 or more was considered significant (*P*<0.05). 

## Results

***Physicochemical properties of honey***

Some physicochemical properties of three monofloral types of honey including thyme, astragalus, and lotus honey, were investigated. The pH, total acidity, and net absorbance are presented in Table 1. The pH of honey was not significantly different between the species (*P*<0.05). Free acidity values ranged from 16 to 26 meq/kg. The acidities of thyme and astragalus were significantly higher than that of lotus honey (*P*<0.05). It has been reported that high free acidity values may indicate fermentation of honey sugar by yeasts, which eventually leads to production of acetic acid. This is likely to be the case with older samples. Also, the net absorbance (A_450_–A_720_) of lotus honey was significantly higher than those of others (*P*<0.05). 

Absorbance at 450 nm is generally related to the presence of pigments, such as carotenoids and flavonoids, which have anti-oxidant properties (15, 20). However, absorbance at 450 could be related to specific contaminating pigments arising from handling, processing, and storage and biochemical reactions (20). Based on these possibilities, the presence of flavonoids was first determined and then the net absorbance was measured in samples. The net absorbance (A_450_–A_720_) of honey solutions varied from 0.25 AU for the lightest honey (astragalus) to 0.45 AU for the darkest honey (lotus). Based on the color and net absorbance assay, lotus honey could have the highest anti-oxidant properties. 

Moreover, as can be seen from Table 1, the total alkaloid value of lotus honey was significantly higher than those of the other two types (*P*<0.05). 

***DPPH radical scavenging activity***

**Figure 1:**
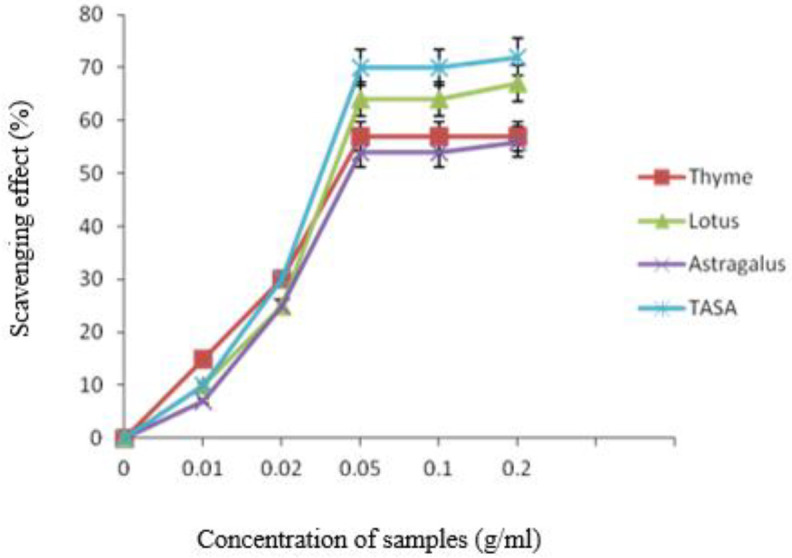
DPPH scavenging effect by honey samples and total alkaloid extract of local *Sophora alopecuroides* (TASA)

The anti-oxidant activity of honey samples was calculated as the percentage of DPPH discoloration. The results are presented in Figure 1A higher radical scavenging effect was observed in lotus honey (the darkest honey) and TASA. The scavenging effect was enhanced by increasing the honey concentration up to 0.05 g/ml. As can be seen from Figure 1, the highest DPPH radical scavenging effects were 54%, 57%, 64%, and 70% in astragalus, thyme, lotus honey, and TASA, respectively. 

***Single and combined MIC of materials and material interaction***

The MIC and FICI results and type of interaction between CIP, honey, and TASA are presented in Tables 2. Three types of honey had similar MICs. Thus, the results are not presented separately for each type of honey, as can be seen in Table 2. The MICs of TASA and CIP alone were similar to previous reports (10). Addition of 30% honey (sub-MIC) significantly increased the susceptibility of M1 strain to CIP (*P*<0.05). The MIC of CIP was reduced by two folds in combination with honey (FICI=0.8). Use of 40% honey also decreased the MIC of tetracycline. In addition, the use of 30% honey caused a four-fold reduction in the MIC of TASA (FICI=0.67). These results indicate that honey may have inhibitory effects on the AcrAB-TolC efflux pump and induce extra effects on the MIC of CIP in the CIP-TASA-honey combination. The synergistic effect of TASA and honey in combination with CIP was confirmed (FICI=0.48). 

***Effect of honey on CIP sensitivity of Escherichia coli***

**Figure 2:**
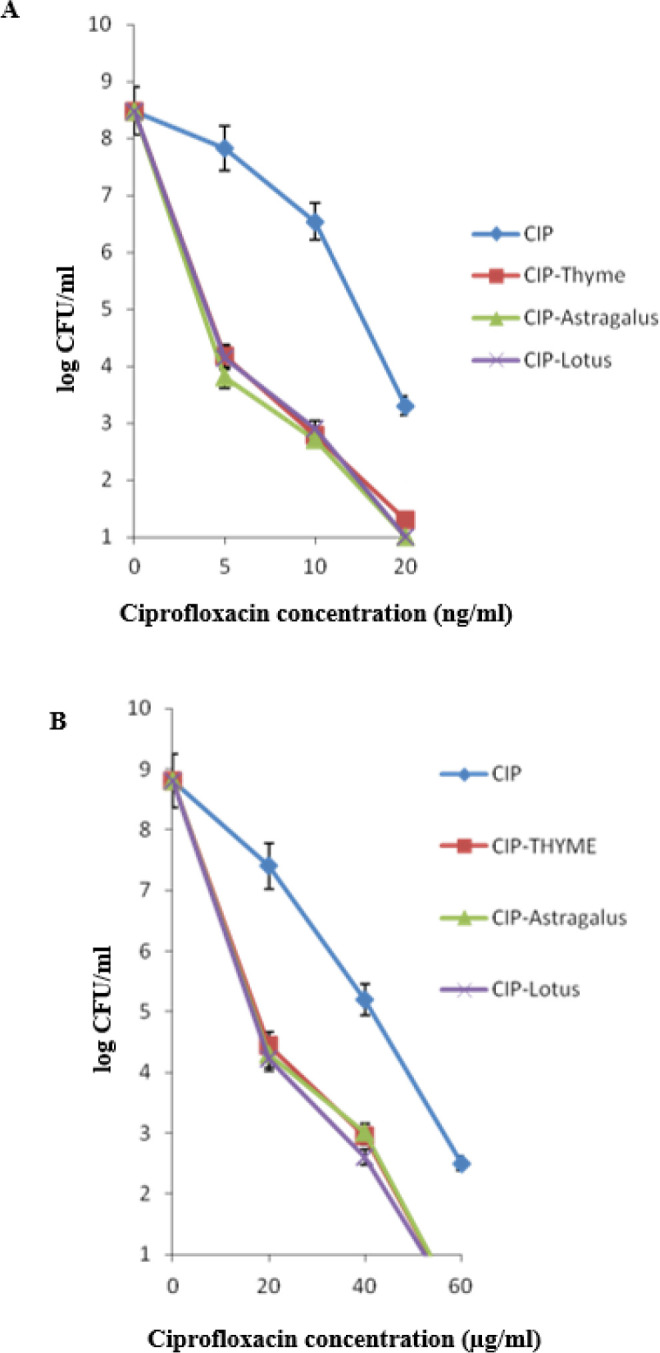
Effect of three types of honey (30% w/v) on the viable counts of MG1655 (A) and M1 (B) in the presence of various concentrations of ciprofloxacin (CIP)

The survival curves of wild type (MG1655) and mutant (M1) strains in the presence of different concentrations of CIP are shown in Figure 2 (A and B). It was found that 20 ng/ml and 60 µg/ml of CIP decreased the colony-forming units (CFUs) by about six log units in MG1655 and M1 strains, respectively. However, in the presence of 30% concentration of honey, both strains showed more susceptibility to CIP. There was no significant difference between the three honey types regarding their effects on survival (*P*<0.05). 

***Expression of acrA and soxS genes***

The three honey types produced similar results in different treatments. Therefore, the results of gene expression for only one honey sample (lotus) are presented in Table 3. The *acrA* gene was overexpressed in the M1 strain following treatment with CIP-honey combination, whereas the level of *acrA* expression was significantly lower than that of CIP-treated M1 (*P*<0.05). A similar result was obtained for *acrA* expression in the M1 strain treated with CIP and TASA in comparison with M1 treated with CIP alone (10). However, expression of this gene reduced significantly to the level of the wild-type strain following treatment of M1 with CIP, honey, and TASA (*P*<0.05). 

It was found that expression of *the soxS* gene increased after exposure to CIP (unpublished data). In the present study, the expression of the *soxS* gene was measured to determine whether treatment of cells with CIP-honey and CIP-honey-TASA could change the expression of this gene. The results (Table 3) indicated that the expression of this gene was still high in CIP-honey-treated cells. However, treatment with CIP-honey-TASA significantly decreased expression of the *soxS* gene in M1 (*P*<0.05). 

## Discussion

Excessive use or misuse of antibiotics, such as CIP, is known to increase the number of MDR strains with an overactivated efflux pump. Alternative therapies using natural products, either alone or in combination with antibiotics have increasingly drawn the researchers’ attention. One of these natural products is honey, which has been used as a topical dressing (wound healing) since ancient times (8). Honey has inhibitory effects on the growth of many microorganisms associated with diseases or infections, including MDR strains (7, 8). 

Honey has synergistic effects with oxacillin, tetracycline, imipenem, and mupirocin against the growth of MRSA strains (8). Its antibacterial activity against *E. coli* has been also investigated in previous studies (3, 8). Use of honey neither causes resistance nor compromises the efficacy of other antimicrobials (8). Therefore, in this study; we aimed to study the inhibitory effects of three monofloral types of honey on highly CIP-resistant *E. coli* strains (M1), either alone or in combination with CIP and TASA. It was previously found that this mutant (M1) has MDR phenotypes as it is resistant to CIP, tetracycline, and chloramphenicol and shows increased AcrAB-TolC efflux pump activity (10). 

All three honey samples were found to have an acidic character. The low pH value of honey inhibits the growth and proliferation of microorganisms. These values were similar to those reported previously worldwide (2). Free acidity values did not exceed the limit (50 meq/kg). It has been reported that high free acidity values may indicate fermentation of honey sugar by yeasts, which eventually leads to production of acetic acid. This is likely to be the case with older samples (2). Thus, all honey samples were fresh. It was found that lotus honey (the darkest honey) had the highest alkaloid value, anti-oxidant activity, and net absorbance (flavonoid compounds). This result is consistent with previous findings obtained for the darkest honey (1, 2). 

We found that all three types of local monofloral honeys similar to other honeys worldwide could inhibit growth of the* E. coli* wild-type strain. They similarly inhibited growth of CIP-resistant mutant (M1). The main antibacterial compounds of commercial honey are hydrogen peroxide and non-peroxide materials, such as phenolic acids and flavonoids with anti-oxidant activities. It seems that the harmony between these compounds results in suitable antimicrobial effects (3, 4). 

It was reported that anti-oxidant compounds, such as glutathione and ascorbic acid, act as protectants for MG1655 against CIP and reduce sensitivity to this antibiotic (19). However, it was found that honey (30%) in combination with CIP increased the sensitivity of wild-type and M1 strains to CIP; this finding may be related to the presence of hydrogen peroxide in honey. It is known that hydrogen peroxide promotes oxidative stress in *E. coli* (9, 21). Oxidative stress is also induced by treatment of bacterial cells with antibiotics such as CIP (9, 22). It was found that there is an additive interaction between CIP and honey on MIC (FICI=0.8). This kind of interaction may result from interaction of CIP with honey on induction of oxidative stress. 

Furthermore, in response to an increased flux of ROS, such as hydrogen peroxide and superoxide anion in *E. coli,* a group of anti-oxidant defense genes is induced by OxyRS and SoxRS regulons (22). In another study, it was found that hydrogen peroxide (0.1˟MIC) increased expression of the *soxS* gene in an *E. coli* mutant resistant to hydrogen peroxide (MIC=10 mM) significantly more than that induced by CIP (0.1˟MIC) in M1 strain (unpublished data). 

In the present study, honey (30%) in combination with CIP led to overexpression of *the soxS* gene, although it was less than CIP alone in M1. This may be related to the low amount of hydrogen peroxide in honey and low permeability of hydrogen peroxide across the *E. coli* membrane (23). Moreover, a previous study revealed that the sub-lethal dose of medical-grade *Leptospermum* honey up-regulated the level of genes involved in stress responses, such as oxidative damage response (4). This result is consistent with our finding, which showed that a combination of honey and CIP enhanced the expression of *soxS*. 

SoxS, similar to MarA, is a regulator of the expression of the AcrAB-TolC efflux pump. It was found that an increase in the expression of the *soxS* gene following treatment of M1 with CIP and honey led to overexpression of the* acrA *gene, but not as much as CIP alone. In a previous study, it was found that the combination of CIP with TASA elevated the expression of *acrA*, although it was less than that induced by CIP alone. Exposure to honey-CIP-TASA significantly reduced the expression of both *soxS* and *acrA* genes (*P*<0.05) in M1 nearly to the level of the wild-type strain. It was suggested that TASA has anti-pump activities (10). The presence of alkaloid compounds in honey was also indicated in this study. The partial synergistic interaction between honey and TASA (FICI=0.67) may suggest their interaction in the decrease of *acrA* expression; in fact, inhibition of the AcrAB-TolC pump eliminated the MDR phenotype. This finding is also consistent with our results, which showed the additive interaction between honey and tetracycline (FICI=0.96). 

Moreover, based on our results, flavonoids and carotenoids are present in honey samples. The high scavenging effect of lotus honey and TASA may imply that in the presence of these exogenous anti-oxidants, the cells reduce the expression of endogenous anti-oxidant genes, such as *soxS*. Additionally, the synergistic interaction between honey and natural products has been previously described. A synergistic interaction was reported for the combination of manuka honey, curcumin, and whey proteins on *E. coli* O157:H7 (24) as well as the combination of black forest honey with garlic juice on *E. coli* ATCC 25922 (25). In the present study, it was found that there are partial synergistic and complete synergistic interactions in honey-TASA and honey-TASA-CIP combinations against M1, respectively. 

**Table 1:** Average values of three independent repeats obtained in physicochemical analysis of three types of honey

	Thyme	Astragalus	Lotus
pH	4.5	4.7	4.9
Free acidity (meq/kg)	26	24	16
(A_450_ – A_720_) Net absorbance (AU)	0.37	0.25	0.45
Total alkaloids (µg/ml)	40	12	80

**Table 2:** The interaction of honeys and plant extract with ciprofloxacin against bacterial strains

Interaction	Combined FICIs	Combined MICs	Individual MICs	Strain/Clone	Compounds & their combination
-	-	-	0.035/50	MG1655	CIP μ(μg/mL)/Honey(s)%
Additivity	0.8	50/30	100/70	M1	
-	-	-	3/50	MG1655	TCY μ(μg/mL)/Honey(s)%
Additivity	0.96	60/40	120/70	M1	
-	-	-	50/3	MG1655	Honey(s)%/ TASA (mg/mL)
~SYN	0.67	30/3	70/12.5	M1	
-	-	-	0.035/3/50	MG1655	CIP μ(μg/mL)/TASA (mg/mL)/Honey(s)% (w/v)
SYN	0.48	10/1/20	100/12.5/70	M1	

Cip, TASA, SYN, and ~SYN are abbreviations for ciprofloxacin, total alkaloid extract, complete synergistic, and partial synergistic interaction, respectively

**Table 3:** Relative gene expression as determined by real time PCR

Strain/Clone	Treatment	acrA	soxS
MG1655	Not treated	1	1
M1	Subinhibitory concentration of Cip	4.7±0.4	2.8±0.1
M1	Subinhibitory concentration of Cip and honey	2.9±0.2	2±0.2
M1	Subinhibitory concentration of Cip, honey, and TASA	0.7±0.1	1.6±0.1

## Conclusion

Local honeys contain various amounts of alkaloids and flavonoids. The interaction between honey and TASA enhances the anti-pump activity and reduces the oxidative stress response in *E. coli* mutants with a high level of resistance to CIP. Further investigations are required to determine whether a combination of CIP, honey, and TASA can be used for therapeutic purposes. 

## Compliance with Ethical Standars

***Ethics Approval***

This article does not contain any studies with human participants or animals performed by any of the authors. 
